# Non-resolving Pulmonary Consolidation Mimicking Tuberculosis: A Diagnostic Pitfall Revealing Lung Adenocarcinoma

**DOI:** 10.7759/cureus.107023

**Published:** 2026-04-14

**Authors:** Vedant Saxena, Anil Sontakke, Saood Ali

**Affiliations:** 1 Respiratory Medicine, NKP Salve Institute of Medical Sciences and Research Centre and Lata Mangeshkar Hospital, Nagpur, IND

**Keywords:** kras mutation, lung adenocarcinoma, nodular opacities, non-resolving pneumonia, tuberculosis

## Abstract

Pulmonary tuberculosis (TB) remains highly prevalent in endemic regions; however, current international and national guidelines emphasize microbiological confirmation prior to initiation of anti-tubercular therapy (ATT) whenever feasible. Empirical treatment without microbiological confirmation represents a deviation from recommended standards and may contribute to diagnostic delay.

We report a 52-year-old male presenting with chronic cough with mucoid expectoration and progressive dyspnea without fever, hemoptysis, or weight loss. Despite negative sputum acid-fast bacilli (AFB) smear and cartridge-based nucleic acid amplification test (CBNAAT), the patient was empirically initiated on ATT based on clinicoradiological suspicion. Lack of clinical and radiological response prompted further evaluation. High-resolution computed tomography (HRCT) demonstrated persistent consolidation with perilymphatic nodules and interlobular septal thickening. Positron emission tomography-computed tomography (PET-CT) revealed metabolically active pulmonary lesions with mediastinal lymphadenopathy and a suspicious adrenal lesion. CT-guided biopsy confirmed moderately differentiated lung adenocarcinoma. Molecular analysis revealed a KRAS mutation with negative programmed death-ligand 1 (PD-L1) expression.

This case underscores the importance of adherence to diagnostic guidelines, early reconsideration of diagnosis in non-resolving consolidation, and the critical role of timely tissue diagnosis in preventing delays in oncological management.

## Introduction

Pulmonary tuberculosis (TB) remains a major public health burden in India, accounting for a significant proportion of global TB incidence [1]. Despite advances in diagnostic modalities, inappropriate empirical initiation of anti-tubercular therapy (ATT) without microbiological confirmation continues to occur in clinical practice, representing a deviation from established guidelines such as those from the World Health Organization (WHO) and national TB programs [1,2]. Several non-infectious conditions, particularly lung malignancies, may closely mimic TB both clinically and radiologically. Lung adenocarcinoma, especially pneumonic-type or lepidic growth patterns, can present as persistent consolidation with air bronchograms and ground-glass opacities, closely resembling infectious processes [3,4]. Failure to recognize this overlap may result in delayed diagnosis and disease progression. Therefore, non-resolving pulmonary consolidation - especially in smear-negative patients - should prompt early re-evaluation with advanced imaging and histopathological confirmation [5,6]. We present a case of lung adenocarcinoma initially misdiagnosed as pulmonary TB, highlighting critical diagnostic pitfalls and emphasizing the need for guideline-directed evaluation.

## Case presentation

A 52-year-old male presented with a six-month history of cough with whitish (mucoid) expectoration and progressive dyspnea on exertion (Modified Medical Research Council Grade II) for 15-20 days. There was no history of fever, hemoptysis, or significant weight loss. The patient was a non-smoker. Baseline evaluation revealed stable vital parameters with oxygen saturation of 96% on room air. Routine laboratory investigations, including complete blood count and liver and renal function tests, were within normal limits. There was no history of diabetes mellitus, human immunodeficiency virus infection, or occupational exposure to dust or carcinogens.

**Figure 1:**
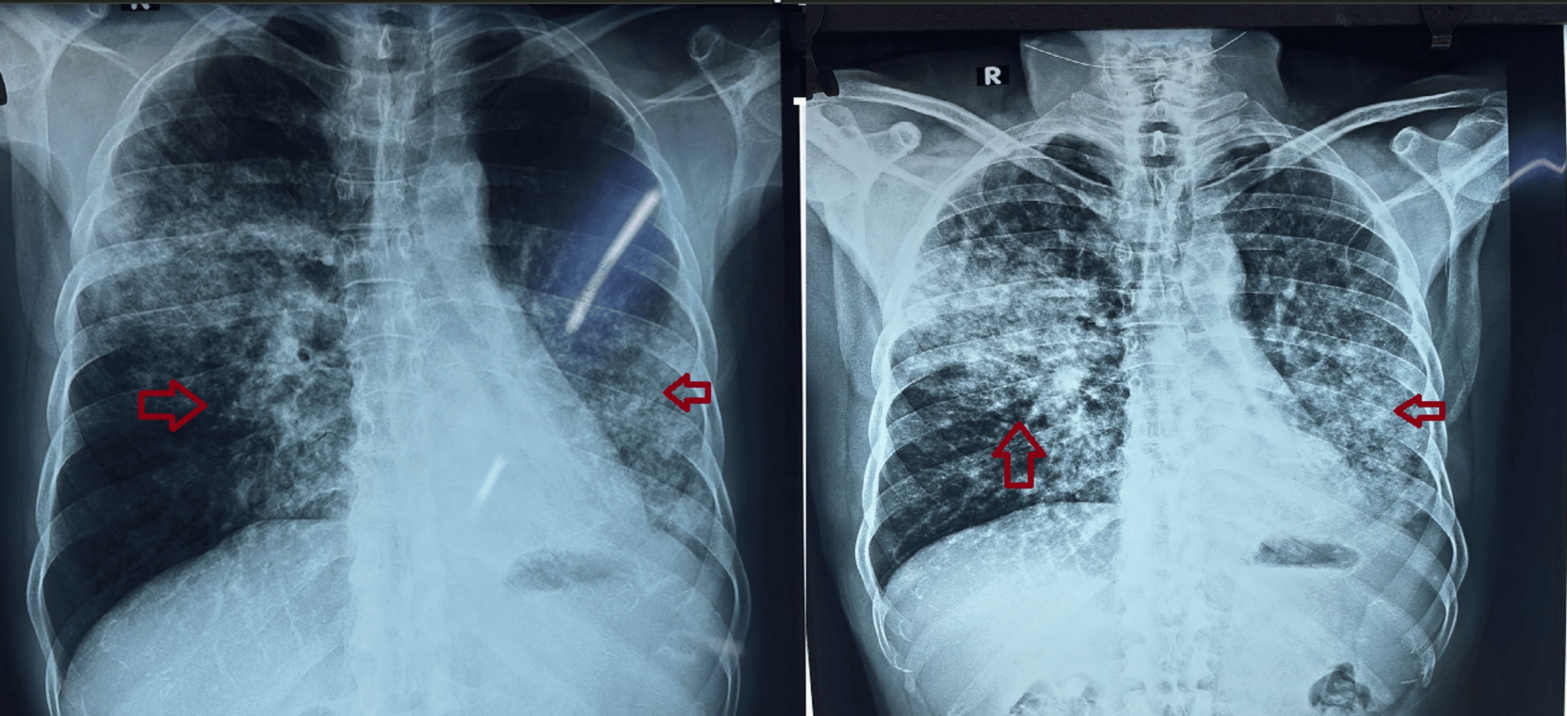
Chest radiograph (posteroanterior view) demonstrating bilateral heterogeneous opacities with predominant involvement of the left lower zone. Follow-up imaging shows persistent bilateral infiltrates with no significant resolution after anti-tubercular therapy.

Despite the absence of microbiological confirmation, the patient was initiated on first-line ATT based on clinicoradiological suspicion. This approach deviated from guideline recommendations, which emphasize microbiological confirmation wherever possible [1,2]. The patient completed approximately six to seven weeks of therapy without symptomatic or radiological improvement. Chest radiography demonstrated persistent bilateral opacities (Figure 1), prompting further evaluation.

Investigations

**Figure 2:**
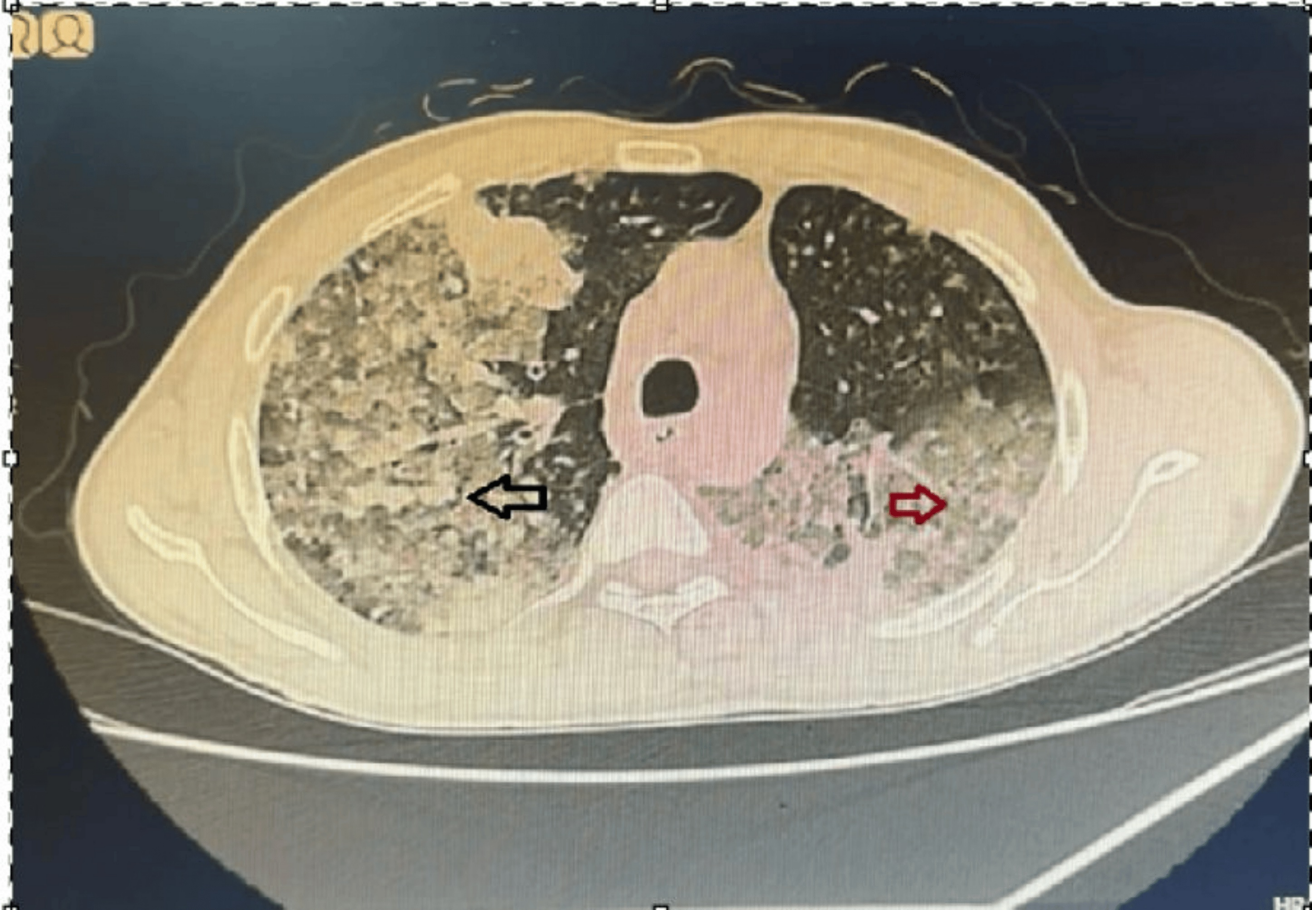
High-resolution computed tomography (HRCT) of the thorax showing dense consolidation with air bronchogram in the left lower lobe, associated with centrilobular and perilymphatic nodules.

Sputum AFB smear and cartridge-based nucleic acid amplification test (CBNAAT) were negative for *Mycobacterium tuberculosis*. Gram stain showed gram-positive cocci in chains; this finding was considered likely colonization rather than clinically significant infection, as there was no clinical or radiological response to antibiotic therapy. Flexible bronchoscopy revealed inflamed mucosa without an endobronchial lesion. Bronchoalveolar lavage cytology was negative for malignancy, consistent with its known limited sensitivity, particularly for peripheral lesions. High-resolution computed tomography (HRCT) of the thorax demonstrated dense consolidation with air bronchograms in the left lower lobe, along with perilymphatic nodules and interlobular septal thickening (Figure 2). These findings raised suspicion for malignancy, although overlap with infectious etiologies is recognized [3,7].

**Figure 3:**
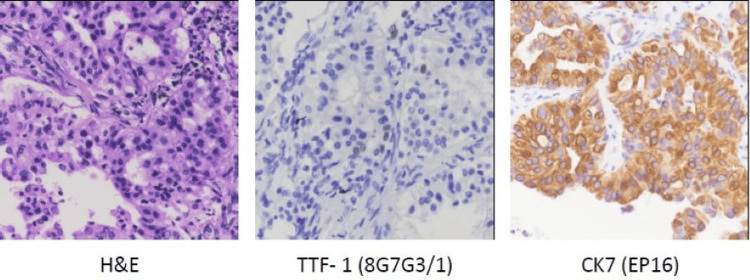
Histopathological section from CT-guided lung biopsy showing tumor tissue arranged in glandular patterns with moderate nuclear atypia, consistent with adenocarcinoma (hematoxylin and eosin stain, ×100 and ×400 magnification).

Further evaluation with positron emission tomography-computed tomography (PET-CT) revealed a metabolically active lesion in the left lower lobe, bilateral mediastinal lymphadenopathy, interlobular septal thickening with nodularity suggestive of lymphangitic carcinomatosis, minimal pleural effusion, and a suspicious right adrenal lesion. Pleural fluid analysis was not performed due to minimal volume. CT-guided lung biopsy confirmed moderately differentiated adenocarcinoma. Immunohistochemistry showed diffuse CK7 positivity and focal TTF-1 positivity, while CK20, CDX2, PAX8, and p40 were negative. This immunoprofile supports a primary pulmonary adenocarcinoma and helps exclude extrapulmonary metastatic sources. Molecular analysis revealed a KRAS mutation with negative programmed death-ligand 1 (PD-L1) expression (Figure 3).

## Discussion

Diagnostic pitfall in TB-endemic settings

Empirical initiation of ATT without microbiological confirmation represents a deviation from established WHO and national guideline recommendations and is considered suboptimal clinical practice, contributing to diagnostic delays.

Clinical red flags against TB

The patient lacked cardinal TB symptoms (fever, weight loss, hemoptysis). Additionally, whitish mucoid expectoration is atypical for TB and should prompt consideration of alternative diagnoses, including malignancy. Lower lobe predominance further represents a radiological red flag, as TB classically involves the upper lobes.

Radiological overlap and differentiation

HRCT findings such as perilymphatic nodules and interlobular septal thickening are characteristic of lymphangitic carcinomatosis but may overlap with infectious processes, including TB, thereby necessitating careful radiological interpretation [3,5,7]. Persistent consolidation with these features should prompt early biopsy.

Role of histopathology

Histopathological confirmation remains the gold standard for diagnosis. CT-guided biopsy provided a definitive diagnosis in this case, underscoring the importance of early tissue sampling [6].

Staging and disease spread

PET-CT findings confirmed metastatic disease, consistent with stage IV classification [4]. Lymphangitic spread is associated with poor prognosis and reflects advanced disease burden [7].

Clinical implications

Failure of radiological resolution within four to six weeks of therapy in smear-negative patients should trigger re-evaluation with computed tomography and biopsy. Prolonged empirical ATT may not only delay diagnosis but also introduce risks such as hepatotoxicity and drug interactions.

## Conclusions

Non-resolving pulmonary consolidation should not be presumed to be TB in the absence of microbiological confirmation. This case highlights the importance of recognizing clinical and radiological red flags, adhering to diagnostic guidelines, and pursuing early histopathological evaluation. Prompt diagnosis is essential to avoid delays in management and improve outcomes in patients with underlying malignancy.
